# Observations on Spasmodic and Convulsive Diseases

**Published:** 1817-05

**Authors:** Richard Moulson


### *Observations on Spasmodic and Convulsive Diseases.*

By Richard Moulson, M. D.

HAVING frequently been baffled in my attempts to cure spasmodic and convulsive diseases by purgatives, and having lost one or two children, when treated according to the principles laid down by the most celebrated practitioners who have written upon the subject, I was determined, the first opportunity that offered, to investigate anatomically the nature of these diseases, in hopes that a more successful practice might be deduced from appearances on dissection. Soon after these failures, a case occurred in the practice of my friend, Dr. Sanders of Edinburgh, which being proof against any internal remedies, I carefully noted down the different muscles spasmodically affected, that I might see whether any difference existed between them and those that were free from spasm. Permission being obtained to examine the child's body, the contents of the abdomen and thorax were found perfectly free from disease, but upon carefully examining the brain and spinal marrow, there appeared sufficient evidence to account for the spasmodic contractions, the cause of the child's death. The nerves distributed to the muscles previously noted down (whether proceeding from the brain or spinal marrow) were found to have at their *origins*,* the blood-vessels preternaturally turgid with blood, whilst the blood-vessels ramifying upon the nerves distributed to muscles free from disease were perfectly natural. These appearances being put to paper, and reasoned upon at leisure, an opinion was formed, that could a sufficient quantity of blood be abstracted from those parts labouring under this turgescence, the disease would be removed. After this dissection, a case of chorea occurred in a girl about 14 years of age, and the purgative plan was prosecuted to the fullest extent: instead of the symptoms being alleviated by this treatment, they were aggravated, insomuch that she was obliged to be held down in bed by her father to prevent her from injuring herself. In lieu of this treatment, leeches were applied to the spine, followed up by blisters and frictions along its whole course, and by these means she completely recovered in a very few days. This

* By the word origin is here meant, that part of the brain or spinal marrow whence a nerve is seen to emerge immediately upon removing its membranes.

is only one of the many examples I could adduce of cases in which the purgative plan failed, and which were relieved by the treatment above mentioned. Having found that abstraction of blood from as near as possible to the origins of the nerves distributed to muscles spasmodically affected had the effect of alleviating the disease, and that a cure was completed by blistering and friction; a case occurred in which I was determined to employ them without the aid of medicine.—A boy, about eleven years of age, was playing with his school-fellows, when suddenly he fell upon the ground in convulsions. When I first saw him, he had been an hour convulsed; the pupils of his eyes were widely dilated, mouth firmly closed, and his hands clenched. I immediately ordered eight leeches to the nape of the neck, desired that as much blood should be obtained as was possible, by the assistance of cloths wrung out of warm water; and that afterwards a large blister should be applied to the part. At two o'clock, P. M. I saw him first, when the leeches were ordered; at six, P. M. the blister was put on; and at ten, P. M. he was free from convulsions, and spoke quite rationally to his parents. The next day he was walking about the house, when I ordered him a laxative; and the third day from his attack I saw him flying a kite in the street. To enumerate cases of this kind, I think unnecessary; but must observe, that in every case of convulsions that has terminated fatally, I have invariably found turgescence at the origins of the nerves distributed to those muscles that were affected. That what is above stated respecting the sanguiferous system in spasmodic diseases is not new, may be seen from the writings of Galen, Alexander Trallianus, Lieutaud, Morgagni,* Burserius, &c.; but they not having drawn any conclusions whereby the practice might be benefited, I have been led to consider, that by seriously attending to this state of the blood-vessels, much trouble and anxiety may be spared the medical attendant, and much suffering to the patient.

The very important observations which have lately been made respecting the sanguiferous in relation to the nervous system by different writers, particularly those by Dr.

---

* In his de Sedibus et Causis Morborum, lib. i. epist. 10, is the relation of the dissection of a young man where the blood-vessels at the origins of the nerves distributed to the superior extremities that had been convulsively affected, were found to be exactly in the state I have described.

Parry, in his Elements of Pathology and Therapeutics,* clearly point out, that more rational deductions of diseases must follow, and that the suppositions of theorists must give way to facts founded upon anatomy, and established by practice. Indeed, such a connexion exists between the vascular and nervous systems, that the one predominating, disease and irregular actions will take place: and it is only when an equilibrium between the two systems is perfectly maintained, that all the functions of the human body proceed in the execution of their office with the greatest regularity. That such is the case, may be elucidated by numerous affections in the human body: in phrenitis, for example, the stimulus of the sanguiferous system produces the most furious delirium, which is so greatly alleviated by depletion, &c.; and here, it may be remarked, that such violent commotions arise from arterial blood; whereas, when venous turgescence takes place and stupor occurs, it is evident that the nervous energy gradually is diminished, and at length ceases, as in apoplexy. When such a column of venous blood presses upon the brain or spinal marrow as not entirely to overcome the nervous energy, then convulsions occur, which I have seen take place in the healthiest persons. I well remember in one young person of a very spare habit of body, who, after dinner, reclined his neck on the chair back, fell asleep, and in an hour afterwards, found himself lying on the floor, at some distance from where he had sat, and the table and chair upset by him. That he had a narrow chance of his life, and owed it entirely to his falling on the floor, was evident the following morning, from the ecchymosis all over his face and neck. So numerous are the causes capable of exciting convulsions, that it may be asked, how will what has been stated above account for worms, &c. in the alimentary canal occasioning them? That they do so, is known to every one; and to explain

---

* In section 744 of Dr. Parry's invaluable work, is remarked, "That chorea owes its orgin to some affection of the brain, is probable, because it is increased by mental agitation, and, for the most part, ceases during sleep. How far this disorder, in all cases, depends on excessive impulse of blood in the brain, it is difficult to ascertain; but it seems occasionally to depend on that cause, since it is aggravated by whatever increases the action of the heart." That the brain, in all cases, is not affected, I have found upon dissection; and have seen verified the words of Lieutaud, which ought never to be forgotten in attendance upon a convulsive disease, viz. "Que la moëlle de l'épine étoit le siége des convulsions qui laissoient la liberté des sens et celle de la parole."—*Précis de la Médecine Pratique.*

how, we must be contented with a little theory, which, whether incorrect or not, appears plausible on beholding the parts of a dead subject. Believing that every sensible part of the human frame is beset with nerves, I can suppose, that any irritation applied to such a part will (if I may be allowed the expression) occasion such a part to exert all its nervous influence to overcome or free itself from such an irritation; of course, an increased quantity of blood will be solicited to support these actions; which actions, too long continued, destroying the usual contractile power of the vessel or vessels, will occasion such a debility in them, as to allow of a turgescence of blood here and there, unequally pressing upon the nerves, thereby occasioning these spasmodic or convulsive movements.*

In the prosecution of some experiments, I had an opportunity of examining a horse that had died suddenly of what was termed the gripes: I could discover no visible cause of death either in the abdomen or thorax; but upon opening the spine, I was astonished to find the blood-vessels all along the spinal marrow, and particularly those distributed upon the origins of the nerves going to the intestines, *gorged* with blood; the vessels of the brain were turgid, but in an inferior degree. I think there can be little hesitation in saying, that could a sufficient quantity of blood have been abstracted from the head, and from along the whole course of the spine, followed up by blistering and frictions, without even further interference, the horse would have had a good chance of recovery. This assertion arises from my having put such remedies into execution with success in cases of convulsions, without the aid of an internal medicine. But it must not be forgotten, that in all cases of spasms and convulsions, originating from worms, sordes, &c. in the alimentary canal, before any alleviation of symptoms can be expected, they must first be removed, and a continuation of purgatives, after their expulsion, can answer no good end, but may prove prejudicial. So long as the intestines retain their full power of peristaltic motion, I cannot conceive that any foreign bodies can be lodged there; for in the practice of those physicians who uniformly administer the oxyde of zinc in cases of chorea, and who have regularly

* The theory I have here advanced, I promise to put to the test of experiment the first opportunity; at present, my time is so fully occupied, that I fear I shall not be able to put it into execution until the summer.

effected a cure by it, it is very probable that many cases must have originated from worms, which have been expelled so soon as the equalization of the circulation of the blood had been procured by the nausea excited by the zinc, whereby the regular actions of the intestines have been established. The favourable result of fever succeeding to convulsions has been remarked from the days of Hippocrates, which good can only arise from the equal distribution of the blood over the whole body; whilst, on the contrary, convulsions succeeding to fever are inauspicious. Whatever internal remedies may be employed tore-move spasmodic affections, they perform what is required of them, either by equalizing the circulation, or by removing the exciting cause; and should they have been continued for a length of time, and the exciting cause removed, still, in many cases, the cure will be incomplete, until the regular functions of the part be restored. The connexion existing between the nerves of one part of the body and another is so complete, together with the pia matral covering continued to their very extremities, that it is easy to account for disorders being so readily communicated from the extremities to their origins. Ever keeping in mind, that, when the function of a part is disordered, and not its structure, we are to look to the *moving powers* in curing or alleviating the complaint; I say, ever keeping in mind that, so soon as our efforts in removing an irritating cause are effected, and the disease continuing, our resources then must be directed towards the moving powers of the part demanding attention. That, remove the cause originally producing the disease, and the effects will cease, does not always hold good, is clearly evinced in many cases of chorea. For instance, should a case of the kind, which has existed for several months, originating from worms or dentition, come under the cognizance of a medical man, the simple removal of the exciting cause will not effect a cure, should the blood-vessels of the nerves of the parts affected have partaken of the general derangement, and a cure will not be obtained until the several parts regain their natural functions. This I have seen exemplified where the vegetable purgatives have been given to their fullest extent, and when they have even aggravated the disease; but, certainly, fewer cases of the kind where calomel has been persevered in; which can only be accounted for from the power which it possesses of equalizing the circulation, and in many instances inducing symptomatic fever; or why would not the other purgatives have answered the same end?

If in convulsive diseases we remove the exciting cause, and still the functions of a part remain disordered, its structure being entire, it will clearly appear, that the indiscriminate employment of purgatives must, by the irritation they create, increase the malady they were intended to cure; and that, in their stead, our endeavours must be directed to those parts whence the power of performing every action originates.

Cases of chorea are continually published, where purgatives have been administered for several weeks before any abatement of symptoms; and it is recorded, that although the stools became nearly natural, yet, by continuing the purgatives, they became black and offensive. Reflecting upon such records, I conceive that an unnecessary continuation of the purgatives occasioned those black and fœtid stools, by inducing a morbid secretion from the intestines, and, luckily for the poor sufferers, such a medicine was combined, viz. calomel, as, within a certain time, was capable of equalizing the circulation, which freed them from their complaints and the further necessity of medical interference. Concerning such practice, it is only necessary to remark, that, had reasoning been called into question respecting the moving powers of each part morbidly affected, more rational conclusions would have been drawn as to the speediest and most judicious remedies to be employed; for it is painful to observe, as Cicero justly remarks, "Consuetudine oculorum, assuescunt animi, neque admirantur, neque requirunt rationes earum rerum, quas semper vident."

To those who advocate the continued administration of purgatives in spasmodic diseases, and who pour one dose after another into the intestines until they effect a cure, the words of Alibert may not be unseasonable: "Les purgatifs n'ont point une propriété absolue sur l'économie animale; leurs effets sont relatifs, non-seulement à leurs doses, mais encore au degré de la susceptibilité nerveuse du conduit intestinal." *

Chester, *15th January,* 1817.

---

* I very well remember Dr. Sanders, in his Lectures, stating the case of a young lady, who had taken amazing doses of calomel without purging her: in order to assist its action, a blister was applied to the back, which had the effect of bringing on a smart purging; and so violent was the ptyalism that succeeded, that she suffered very considerably in her health, and several weeks elapsed before she recovered.

